# Identification of Aberrantly Expressed Genes during Aging in Rat Nucleus Pulposus Cells

**DOI:** 10.1155/2019/2785207

**Published:** 2019-07-10

**Authors:** Shi Cheng, Xiaochuan Li, Linghan Lin, Zhiwei Jia, Yachao Zhao, Deli Wang, Dike Ruan, Yu Zhang

**Affiliations:** ^1^Department of Orthopedics, Guangdong General Hospital, Guangdong Academy of Medical Science, South China University of Technology, Guangzhou 510080, China; ^2^Department of Orthopedic Surgery, Gaozhou People's Hospital, Guangdong 525200, China; ^3^Post-Doctoral Innovation Practice Base of Gaozhou People's Hospital, Guangdong 525200, China; ^4^Department of Orthopaedics, The Sixth Medical Centre of PLA General Hospital, 100048 Beijing, China; ^5^Department of Orthopaedics, The 306th Hospital of People's Liberation Army, Beijing, China; ^6^Department of Bone & Joint Surgery, Peking University Shenzhen Hospital, Shenzhen, 518036 Guangdong, China; ^7^National & Local Joint Engineering Research Center of Orthopaedic Biomaterials, Peking University Shenzhen Hospital, Shenzhen, 518036 Guangdong, China

## Abstract

Nucleus pulposus cells (NPCs) play a vital role in maintaining the homeostasis of the intervertebral disc (IVD). Previous studies have discovered that NPCs exhibited malfunction due to cellular senescence during disc aging and degeneration; this might be one of the key factors of IVD degeneration. Thus, we conducted this study in order to investigate the altered biofunction and the underlying genes and pathways of senescent NPCs. We isolated and identified NPCs from the tail discs of young (2 months) and old (24 months) SD rats and confirmed the senescent phenotype through SA-*β*-gal staining. CCK-8 assay, transwell assay, and cell scratch assay were adopted to detect the proliferous and migratory ability of two groups. Then, a rat Gene Chip Clariom™ S array was used to detect differentially expressed genes (DEGs). After rigorous bioinformatics analysis of the raw data, totally, 1038 differentially expressed genes with a fold change > 1.5 were identified out of 23189 probes. Among them, 617 were upregulated and 421 were downregulated. Furthermore, Gene Ontology (GO) and Kyoto Encyclopedia of Genes and Genomes (KEGG) pathway analysis were conducted and revealed numerous number of enriched GO terms and signaling pathways associated with senescence of NPCs. A protein-protein interaction (PPI) network of the DEGs was constructed using the Search Tool for the Retrieval of Interacting Genes (STRING) database and Cytoscape software. Module analysis was conducted for the PPI network using the MCODE plugin in Cytoscape. Hub genes were identified by the CytoHubba plugin in Cytoscape. Derived 5 hub genes and most significantly up- or downregulated genes were further verified by real-time PCR. The present study investigated underlying mechanisms in the senescence of NPCs on a genome-wide scale. The illumination of molecular mechanisms of NPCs senescence may assist the development of novel biological methods to treat degenerative disc diseases.

## 1. Introduction

Low back pain (LBP) is a major age-related disease, not only contributing to patients' suffering and disability but also causing large financial burden to society [1]. Intervertebral disc degeneration (IVDD) has been confirmed to be one of the most fundamental pathological changes of LBP [2]. Due to a largely unknown mechanism of IVDD, effective therapy methods still need investigation.

Traditional therapy strategies including surgery and conservative therapy are aimed at alleviating symptoms instead of regenerating the degenerated disc. Thus, biological approaches which mainly focus on restoring the structure and function of the IVD are considered more promising [3, 4]. The structure of the intervertebral disc could be divided into three different regions: nucleus pulposus (NP), annulus fibrosus (AF), and cartilaginous endplate (CEP) [5]. NP is a kind of gelatinous tissue containing extracellular matrix (ECM) comprising highly hydrated proteoglycan, collagen fibers, and aggrecan [6]. It plays a vital role in maintaining the physiological function of IVD because NP could absorb stress when the IVD is confronting diverse mechanical impact [7]. Nucleus pulposus cells (NPCs) are the organ-specific cells residing in the nucleus pulposus [8]. NPCs are responsible for the metabolism homeostasis of the ECM by producing collagen I, collagen II, and proteoglycan, which are the main components of the gelatinous structures of NP [9]. During aging and degeneration of IVD, the normal function of NPCs was disrupted, thus resulting in aberrant metabolism of ECM, which could accelerate the process of IVDD [10, 11]. Cytotherapy by reactivate degenerated NPCs has been proposed to be an ideal biological therapy method to treat IVDD [11]. However, the specific mechanism of NPC degeneration is still unknown, which hindered the development of cytotherapy.

Cell senescence is defined as a cellular program that leads to a stable growth arrest along with distinct phenotypic alterations and presentation of senescence-associated secretory phenotype (SASP) [12, 13]. Senescence of disc cells has been widely accepted as one of the major factors of IVD degeneration and aging [14, 15]. The number of viable cell in NP was decreased, and the cellular function of NPCs is being impaired with age, eventually leading to biomechanical failure and degeneration of IVD [15, 16]. There were two historically forms of senescence: one is replicative senescence which is related to shortened telomere length; the other is stress-induced premature senescence which is induced by a variety of environmental stimuli [17, 18]. Among them, time-dependent accumulation of cell replication and replicative senescence is considered to be more related with aging [19]. Accumulating researches focus on rejuvenating aged NPCs by preventing senescence [11]. However, little progress has been achieved due to the unclear potential regulators or mechanisms of senescence of NPCs. Therefore, elucidating the major regulators and mechanisms underlying NPCs senescence will help us better understand the pathogenesis of IVD aging and degeneration and may illustrate a new therapy target to rejuvenate aged IVD.

Gene microarray technology can simultaneously analyse differences in the expression level of thousands of genes from predefined groups of samples [20]. It also has the advantage of highly effective evaluation of whole genome-wide expression changes [21]. This technology gives researchers a novel point of view to investigate the mechanism of different diseases more deeply. Although the senescence of NPCs plays an important role during IVDD, yet there were limited number of studies that focus on the aberrantly expressed genes during NPC aging.

Thus, the aim of this study was to investigate the abnormally expressed mRNA and signaling pathways during NPC aging by the method of microarray analysis and bioinformatics analysis. We also compared the migration capability between young and old NPCs because decreased migration of IVD cells may be another important reason for the declining regenerative potential of IVD. These analyses may help to elucidate the senescence mechanism of NPCs, which will contribute in identifying the key factors necessary to rejuvenate NPCs in IVDD patients and promoting the effect of cytotherapy in IVDD.

## 2. Materials and Methods

### 2.1. Ethic Statement

All experimental procedures described blow were reviewed and approved by the Laboratory Animal Ethics Committee of The Sixth Medical Centre of PLA General Hospital, Beijing, China, and carried out in accordance with the relevant guidelines and regulations.

### 2.2. Isolation and Culture of NPCs

12 Sprague-Dawley (SD) rats were involved in this experiment and were divided into two groups according to their age: the young group (*n* = 6, 2 months old) and old group (*n* = 6, 24 months old). 24-month-old rats were defined as the old group according to a previous study [22]. Then, they were sacrificed by intraperitoneal injection of 5 ml 10% chloral hydrate. After being soaked in 70% ethanol for 1 h, the coccygeal NP tissues (C3-C7) of each rat were collected by ophthalmic surgical instruments under a sterile dissecting microscope. After being washed with PBS for three times, the harvested NP tissues were mechanically minced and digested with 0.2% collagenase II (Sigma-Aldrich, St. Louis, MO, USA) in Dulbecco's modified Eagle's medium-low glucose (DMEM-LG, Solarbio Science & Technology Co. Ltd., Beijing, China) for 4 h. The suspension was centrifuged at 1000 rpm/min for 5 min. Then, the suspension solution was discarded, and the pellets were resuspended with standard culture medium containing DMEM-LG, 10% FBS (Gibco BRL, Grand Island, NY, USA), and 1% penicillin-streptomycin (Hyclone, USA). Finally, the cell pellets were cultured in 25 cm^2^ cell culture flasks in a humidified incubator at 37°C and 5% CO_2_. The culture medium was replaced every two days. When the cells reached 70-80% confluence, they were collected using 0.25% trypsin-EDTA (Gibco, USA) and subcultured at 1 : 3; cells at passage 2 were used for experiment.

### 2.3. Cell Phenotype Identification

To identify the cell phenotype of young and old NPCs, a series of surface markers were detected. Both groups of NPCs at P2 were collected and resuspended in cold PBS to a concentration of 1 × 10^6^ cells/ml. Then, 100 *μ*l cell suspension was incubated with antibodies against CD34-PE, CD24-FITC, CD29-PE, CD45-FITC, and CD90-PerCP-Cy5.5 (Abcam, Cambridge, MA, USA) for 30 min at 4°C in the dark. Then, cells were washed twice and resuspended in 500 *μ*l PBS. Finally, quantitative analyses for the expression of surface markers of each samples were performed using the FACSCalibur system (FACScan, BD, USA).

### 2.4. Senescence-Associated *β*-Galactosidase (SA-*β*-gal) Staining

When NPCs were cultured to 80%-90% confluence, SA-*β*-gal staining (Beyotime Institute of Biotechnology, China) was performed to analyse the rate of senescent cells in the young and old groups according to the manufacturers' protocol. Briefly, cells were washed by PBS for three times, then fixed with fixative solution for 15 minutes at RT, washed twice with PBS, and stained with X-Gal containing SA-*β*-gal working solution overnight at 37°C without CO_2_. Quantification analysis was performed under fluorescence microscopy by determining the average percentage of total SA-*β*-gal-positive cells in 5 randomly selected fields of each well.

### 2.5. Cell Counting Kit-8 Proliferation Assay

To compare the proliferation ability of young and old NPCs, P2 cells were plated at a 96-well plate at 1000 cells/well and cultured in standard medium containing 10% FBS for 1, 3, 5, 7, and 9 days. At each time point, the medium was replaced by the mixture solution which contains 100 *μ*l fresh medium and 10 *μ*l CCK-8 reagent. After being incubated in the humidified incubator at 37°C and 5% CO_2_ for 4 h, the absorbance of each sample was measured at 450 nm using a microplate reader (Model 680, Bio-Rad Laboratories K.K., Tokyo, Japan).

### 2.6. Cell Scratch Assay

Young and old NPCs were seeded in 6-well plates at a concentration of 1 × 10^5^ cells/ml. After growing to 100% confluence, parallel scratches were made at the bottom of the 6-well plate with a 200 ml pipet tip. The suspended cells were washed off with serum-free medium, and the width of the scratches was observed and pictured at 0 h and 24 h under an Olympus photomicroscope. The migration of the cells was determined by measuring the distance between the wound edges using ImageJ software. Three different areas were measured for each group, and the average distance was calculated for analysis.

### 2.7. Transwell Assay

The migration capability of young and old NPCs was evaluated with transwell cell culture chambers (pore size 8 *μ*m, Corning, USA), and these chambers were inserted into 24-well plates. The lower chamber was filled with 600 ml medium with 10% FBS, and the upper chamber contained 150 *μ*l of DMEM along and 5 × 10^4^ cells. After 24 h, the media in both chambers were removed and the nonmigratory cells in the upper chamber were wiped off gently by a cotton swab. After being fixed with 4% paraformaldehyde, the migrated cells in the lower chamber were stained with 0.1% crystal violet at room temperature for 30 min. Then, cells traversing the membranes were counted in three randomly selected areas under a light microscope (Olympus Optical Co. Ltd., Tokyo, Japan) at 100x magnification.

### 2.8. RNA Extraction and Quality Control

After being grown to 90% confluence, NPCs of the young and old groups were treated with Trizol. Then, the total RNA was extracted and purified with an RNase Kit (Bio-Rad, CA, USA). The quality of derived mRNA was measured by a spectrophotometer (NanoDrop-1000, Thermo Scientific, MA, USA). The mRNA integrity and DNA contamination were detected by agarose-gel electrophoresis (results are shown in supplementary materials (available here)).

### 2.9. Microarray Analysis

Obtained RNA from young and old NPCs was hybridised to Rat GeneChip Clariom™ S Array from Affymetrix Corporation. The procedures of hybridization and scanning of the microarray were performed according to Whole Transcript (WT) Expression Arrays User Guide of Affymetrix Corporation. Briefly, after the process of probe set signal integration, background correction, and quantile normalisation, these files were transferred to the Affymetrix Transcriptome Analysis Console software to analyse the differentially expressed genes (DEGs). The threshold set for up- and downregulated genes was fold change ≥ 1.5 and *P* < 0.05. The data had been uploaded to the NCBI Gene Expression Omnibus (GEO) and can be accessed via GEO Series accession [GEO:GSE126883] (https://www.ncbi.nlm.nih.gov/geo/query/acc.cgi?acc=GSE126883).

### 2.10. GO Functional and KEGG Pathway Enrichment Analysis

The Database for Annotation, Visualization, and Integrated Discovery (DAVID, http://david.abcc.ncifcrf.gov/) is a gene functional classification implement that accommodates a set of functional annotation tools for investigators to analyse the biological roles of genes and to perform GO (Gene Ontology) and KEGG (Kyoto Encyclopedia of Genes and Genomes) pathway enrichment analysis of DEGs. The functions and pathway enrichment of up- and downregulated DEGs were analysed using the DAVID database. A count > 2 and EASE > 0.1 was considered as the cut-off criteria.

### 2.11. PPI Network Construction and Module Analysis

Functional PPI analysis was essential to interpret the molecular mechanisms of key cellular activities. The Search Tool for the Retrieval of Interacting Genes (STRING, https://string-db.org/) database was adopted to obtain the PPI relationships for DEGs. Briefly, DEGs were uploaded to the STRING database, and the result which interaction score is more than 0.7 (high confidence) was visualized in Cytoscape software. Furthermore, significant modules were detected through the MCODE (Molecular Complex Detection) plugin in Cytoscape based on the constructed PPI networks with the criteria of *K* score = 4, Degree cut − off = 2; node score cut − off = 0.2, and maximum depth = 100. GO functional and KEGG pathway enrichment analyses of the highest score module were performed using DAVID.

### 2.12. Identification of Hub Genes

Cytoscape software was applied to analyse the hub genes, which are important nodes with many interaction partners. We utilized the CytoHubba plugin in Cytoscape to find hub genes and employed six calculation methods: Degree, EPC, EcCentricity, MCC, BottleNeck, and MNC. The intersecting genes derived using these six algorithms represent key candidate genes with important biological regulatory functions. These hub genes were further performed by GO and KEGG pathway enrichment analyses using the DAVID database.

### 2.13. Real-Time PCR

To validate the microarray results, derived 5 hub genes and the most significantly up- or downregulated genes were selected for the real-time PCR validation. Briefly, complementary DNA (cDNA) was synthesized by the reverse transcript using the Quantscript RT Kit (TianGen Biotech, China) according to the manufacturer's protocol. Then, derived cDNAs were taken for quantitative real-time PCR (qPCR) using a SYBR Premix Ex Taq™ (Tli RnaseH Plus; TaKaRa Bio, Otsu, Japan) in a Peltier thermal cycler (Bio-Rad Laboratories K.K., Tokyo, Japan) at an ultimate reaction volume of 20 *μ*l. The cDNA was amplified for 40 cycles. GAPDH was selected as the internal control to calculate the relative expression of target genes by the 2^-*ΔΔ*CT^ method. All reactions were performed in triplicate, and the sequences of the used primers are shown in Table 1.

### 2.14. Statistical Analysis

All the data were expressed as the means ± SD. The comparative analyses between the groups were made by independent sample *t*-tests to determine the significant difference through SPSS 20.0 software (Chicago IL, USA). A chi-square test was adopted for enumeration data. *P* < 0.05 was considered statistically significant.

## 3. Results

### 3.1. Immunophenotypes of Young and Old NPCs

**Figure 1:**
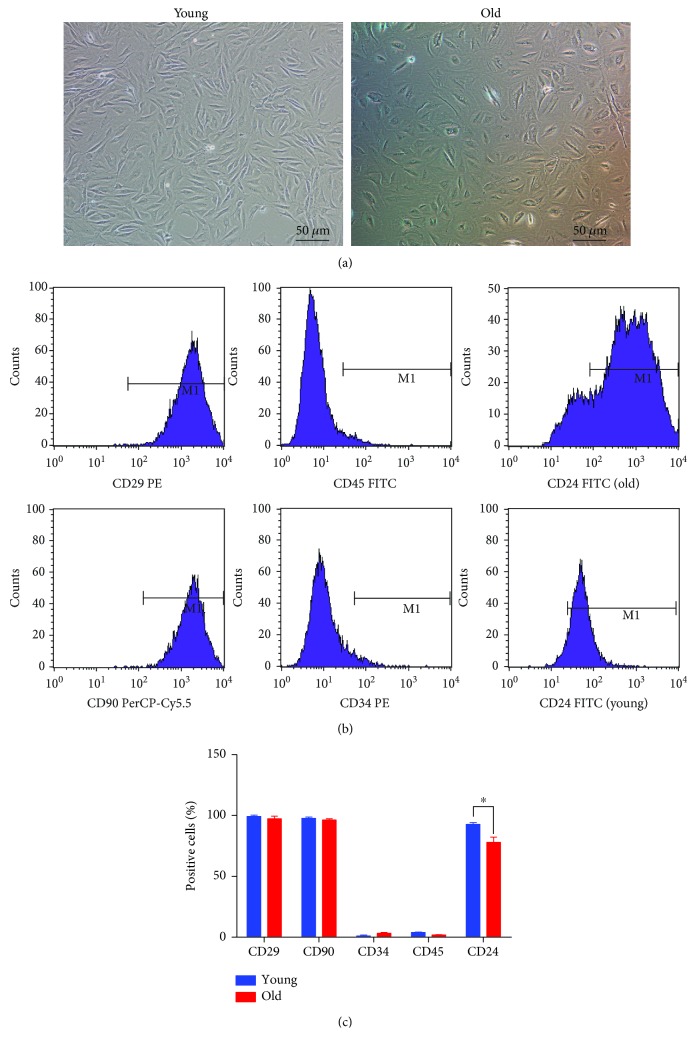
The cell morphology and immunophenotype identification of young and old NPCs. (a) Cell morphology of P2 young and old NPCs. (b, c) Both young and old NPCs exhibited high expression of CD29 and CD90 (>95%) and low expression of CD34 and CD45 (<5%). The expression of CD24 in the young group was 92.68 ± 0.88% and in the old group was 77.91 ± 2.49%. The difference of CD24 expression between young and old NPCs was significant (*P* < 0.05). ^∗^ *P* < 0.05 compared with the young group.

NPCs were successfully isolated from rat nucleus pulposus tissues. The P2 generation of young NPCs exhibited a characteristic of small, spindle-like morphology, with an abundant cytoplasm containing large ovoid and prominent nucleoli. Old NPCs had a more relatively round shape with polygonal and flat morphology (Figure 1(a)).

A series of cell surface antigens were selected to detect the cell surface marker of young and old NPCs [23, 24]. Both groups of NPCs were highly positive for CD29 and CD90 and CD24 (Figures 1(b) and 1(c)). However, the expression of CD24 was significantly lower in the old group than that in the young group (*P* < 0.05). The positive rates in the young group were CD29 (99.15 ± 0.67%), CD90 (97.63 ± 0.62%), and CD24 (92.68 ± 0.88%). The positive rates in the old group were CD29 (97.3 ± 1.26%), CD90 (96.36 ± 0.54%), and CD24 (77.91 ± 2.49%). All NPCs were negative for the hematopoietic stem cell markers CD34 (1.1 ± 0.4% in the young group and 3.98 ± 0.22% in the old group) and CD45 (3.98 ± 0.22% in the young group and 1.9 ± 0.16% in the old group) (Figures 1(b) and 1(c)).

### 3.2. *β*-Galactosidase (SA-*β*-gal) Staining and Aging-Related Decline in Proliferation and Migration Ability

**Figure 2:**
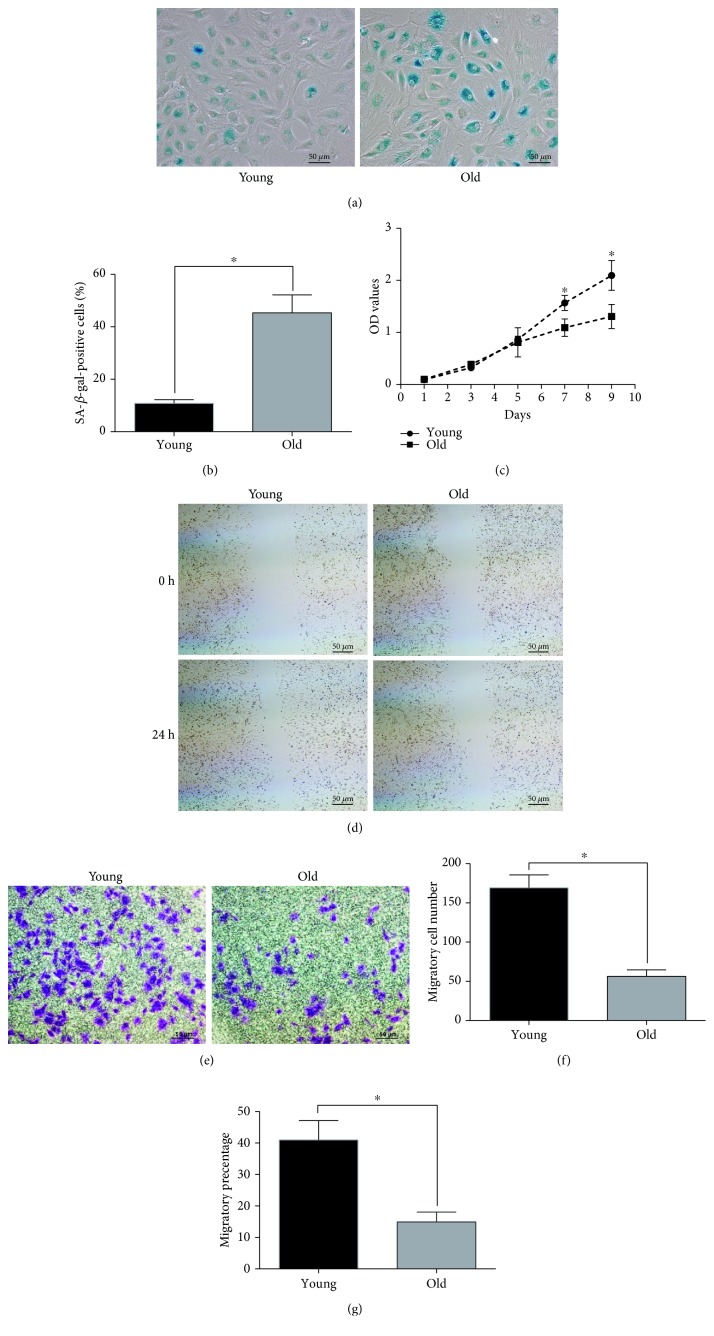
The aging phenotype and migration capacity of young and old NPCs. (a, b) Representative images of SA-*β*-gal staining for the detection of senescent NPCs from the young and old groups. Quantitative analysis showed that the senescent cell number in the old group was higher than that in the young group. (c) Cell proliferation curve detected by CCK-8 assay. The OD value is higher in the young group after 7 days culture, indicating a higher proliferation capability of young NPCs. (d, g) Cell scratch assay showed declined migrated percentage in old NPCs after 12 h. (e, f) Transwell assay showed a declined migrated cell number in the old group. All samples examined in triplicate. Data are presented as the mean ± SD. ^∗^ *P* < 0.05.

*β*-Galactosidase (SA-*β*-gal) staining is a sensitive measurement to detect senescent cells, the results showed a higher positive staining cell percentage in the old group (45.32 ± 6.87%) than that in the young group (10.84 ± 1.41%) (*P* < 0.05) (Figures 2(a) and 2(b)). To assess the proliferation of young and old NPCs, CCK-8 assay was performed. Results showed old NPCs went into early plateau phase approx. 7 days after initially culture and young NPCs did not enter a growth plateau until 9 days of culture (Figure 2(c)). The migration ability was assessed through transwell assay and cell scratch assay. Results of transwell assay showed a decreased migration cell number in the old group (56.33 ± 8.327) compared with the young group cells (169.3 ± 16.44) (*P* < 0.05) (Figures 2(e) and 2(f)). Quantification of the migration area percent after 24 h of the young group (41.02 ± 6.13%) is higher than that of the old group (14.9 ± 3.15%) (*P* < 0.05), indicating a decreased migration speed of old NPCs (Figures 2(d) and 2(g)). Thus, our results clearly demonstrate a dramatic decrease in the proliferous and migratory capacity of NPCs during aging.

### 3.3. Identification of Differentially Expressed Genes

**Figure 3:**
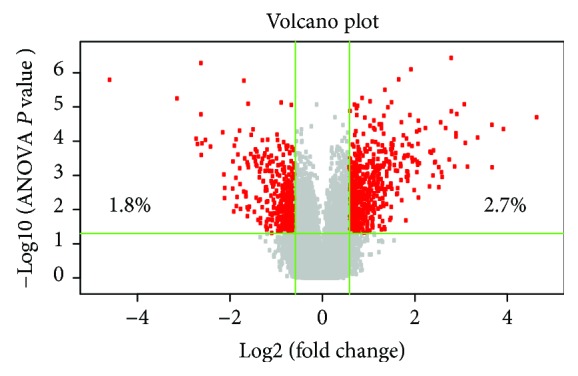
Volcano plot showed differentially expressed genes between the young and old groups. Volcano plot of differentially expressed genes in young and old group. Left: significantly downregulated genes, which account for 1.8% of total detected probes. Middle: nondifferentially expressed genes. Right: significantly upregulated genes, which account for 2.7% of total detected probes (based on ∣log2 FC∣ > 2 and *P* < 0.05). Heat map and hierarchical clustering of DEG profile comparison between the young and old NPCs are included in the supplementary materials.

To detect molecular factors involved in NPC aging, we performed microchip hybridization with RNA from NPCs of the young and old groups. A total of 1038 differentially expressed genes (DEGs) were detected, including 617 upregulated genes and 421 downregulated genes. The upregulated genes refer to those increase expressed in old NPCs. Volcano plot was plotted to show the DEGs of two groups according to the gene expression values (Figure 3). The hierarchical clustering heat map is shown in the supplementary materials. The greatest upregulated gene is kininogen 1 (fold change = 25.06), followed by lipocalin 2, upregulated, and EGF-containing fibulin-like extracellular matrix protein 1, upregulated. The greatest downregulated gene is periostin (fold change = −24.05), followed by neuronal regeneration-related protein, downregulated, and dermatopontin, downregulated. The top 10 up- and downregulated genes are shown in Tables 2 and 3.

### 3.4. GO Functional and KEGG Pathway Enrichment Analysis of DEGs

**Figure 4:**
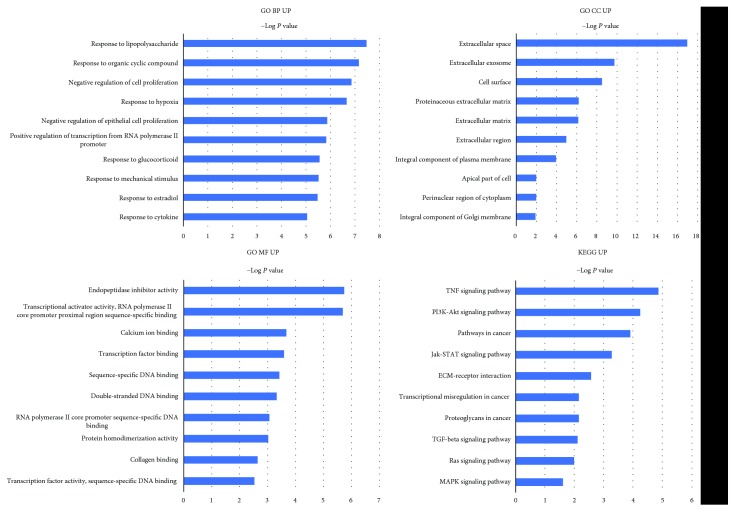
Go function and KEGG pathway enrichment analysis of upregulated genes. Top 5 most significantly enriched GO terms and KEGG pathways of upregulated genes. The length of the bars on the *x*-axis represents the negative logarithm of the *P* value (-log10 *P*) of each pathway; higher (-log10 *P*) values indicate a higher significance, and lower (-log10 *P*) values indicate a lower significance. The GO terms and KEGG pathway names are shown on the *y*-axis. GO MF UP, GO CC UP, and GO BP UP refer to GO terms enriched by upregulated genes. KEGG UP refers to the KEGG terms enriched by upregulated genes.

**Figure 5:**
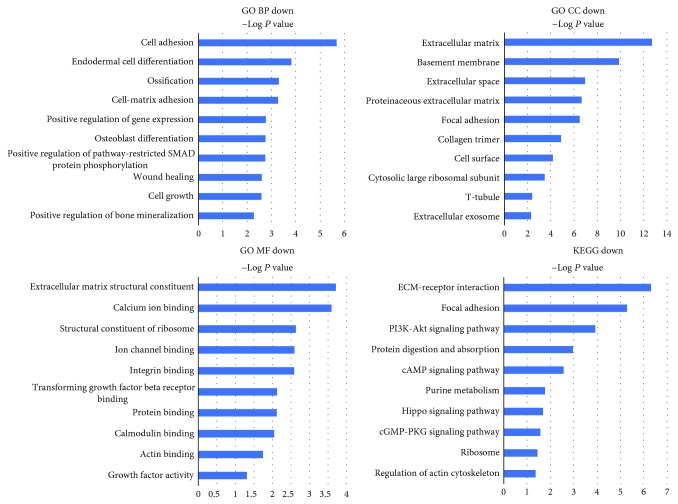
Go functional and KEGG pathway enrichment analysis of downregulated genes. Top 5 most significantly enriched GO terms and KEGG pathways of downregulated genes. The length of the bars on the *x*-axis represents the negative logarithm of the *P* value (-log10 *P*) of each pathway; higher (-log10 *P*) values indicate a higher significance, and lower (-log10 *P*) values indicate a lower significance. The GO terms and KEGG pathway names are shown on the *y*-axis. GO MF DOWN, GO CC DOWN, and GO BP DOWN refer to GO terms enriched by downregulated genes. KEGG DOWN refers to the KEGG terms enriched by downregulated genes.

The functions of up and downregulated DEGs were evaluated at DAVID. The GO analysis showed that, in terms of biological processes (BP), the upregulated genes were mainly enriched in response to lipopolysaccharide and response to organic cyclic compound and negative regulation of cell proliferation (Table 4 and Figure 4). The downregulated genes were mainly enriched in cell adhesion, endodermal cell differentiation, and ossification (Table 5 and Figure 5). In terms of cellular components (CC), upregulated genes were mainly enriched in the extracellular space and extracellular exosome (Table 4 and Figure 4). Downregulated genes were mainly enriched in the extracellular matrix and basement membrane (Table 5 and Figure 5). In terms of molecular function (MF), upregulated genes were mainly enriched in endopeptidase inhibitor activity and transcriptional activator activity (Table 4 and Figure 4), while downregulated genes were mainly enriched in extracellular matrix structural constituent and calcium ion binding (Table 5 and Figure 5).

The KEGG pathway analysis showed that the upregulated genes were enriched in 36 pathways; the most significant pathway was the TNF signaling pathway, and the top 5 significant pathways are shown in Table 4 and Figure 4. The downregulated genes were enriched in 19 pathways; among them, the most significant pathway is ECM-receptor interaction, and the top 5 significant pathways are shown in Table 5 and Figure 5.

### 3.5. PPI Analysis and Hub Gene Screening

**Figure 6:**
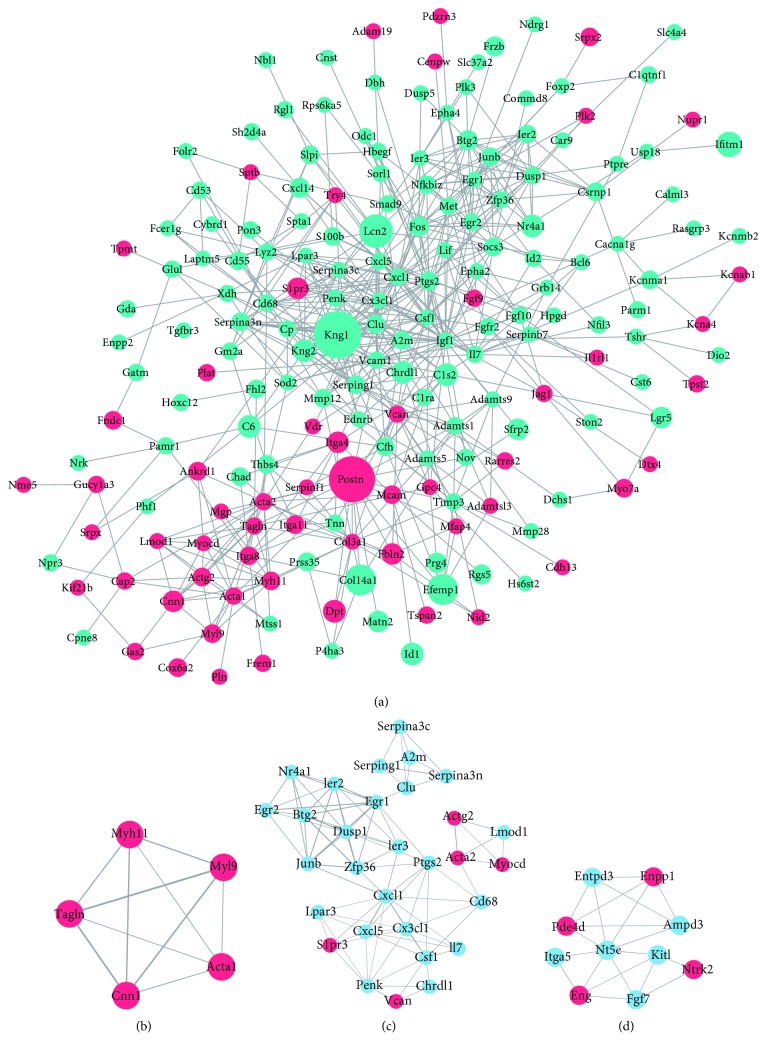
Protein-protein interaction network and significant modules of differentially expressed genes. (a) Red: significantly downregulated genes; green and light blue: significantly upregulated genes. Node size is positively related to fold change; edge width is positively related to the combined score. (b, c, d) Significant modules in the protein-protein interaction (PPI) network with a MCODE score ≥ 4: (b) module 1 (score = 5); (c) module 2 (score = 6.276); and (d) module 3 (score = 4.444).

Based on information from the STRING database, a PPI network comprising 311 nodes and 696 edges with parameters including a minimum required interaction score > 0.7 (high confidence) was constructed using the Cytoscape software (Figure 6(a)). Then, the networks were analysed by plugin MCODE with the criteria of node score > 4 and number of nodes > 4. Finally, 3 significant modules were selected (Figures 6(b)–6(d)). The KEGG pathways enriched of the genes in the highest scored modules (score 6.276) were the TNF signaling pathway, PI3K-Akt signaling pathway, and cytokine-cytokine receptor interaction; the GO biological processes were chiefly enriched in cellular response to organic substance, skeletal muscle cell differentiation, and inflammatory response (Table 6). Then, we detected the hub genes in the network. After running the CytoHubba plugin, there were 5 hub genes identified by the 6 calculation methods (Degree, EPC, EcCentricity, BottleNeck, MCC, and MNC). Results are listed in Table 7. The 6 most significant genes were chemokine (C-X-C motif), ligand 1 (Cxcl1), early growth response 1 (Egr1), FBJ osteosarcoma oncogene (Fos), insulin-like growth factor 1 (Igf1), and prostaglandin-endoperoxide synthase 2 (Ptgs2). Furthermore, we performed GO and KEGG enrichment analysis of hub genes in DAVID. These hub genes were mainly enriched in the TNF signaling pathway and pathways in cancer (Table 8). The chief GO biological processes were in response to lipopolysaccharide and response to glucocorticoid (Table 8).

### 3.6. Validation of Hub Gene by Real-Time PCR

**Figure 7:**
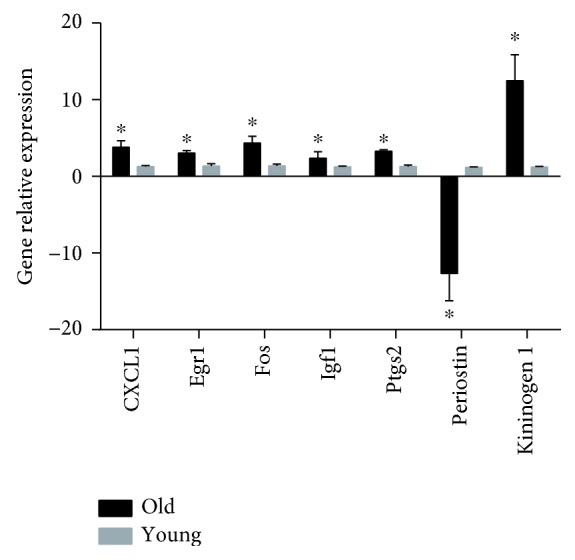
Validation of microarray results by RT-PCR. Five hub genes (Cxcl1, Egr1, Fos, Igf1, and Ptgs2) and the most significantly upregulated (kininogen 1) and downregulated (periostin) genes were selected for the real-time PCR validation. GAPDH was selected as the internal control. Data are presented as the mean ± SD. ^∗^ *P* < 0.05 compared with the young group.

To validate the results of microarray date, we selected the 5 hub genes and the most significantly up- or downregulated genes for real-time PCR analysis. Results were showed consistent with the microarray data. The gene chip analysis demonstrated these mRNAs were upregulated up to 2.643-fold (CXCL1), 5.429-fold (Fos), 2.386-fold (Igf1), 2.369-fold (Egf1), and 3.748-fold (Ptgs2). The RT-PCR results exhibit the expression of CXCL1 (*P* < 0.05), Fos (*P* < 0.05), Igf1 (*P* < 0.05), Egf1 (*P* < 0.05), and Ptgs2 (*P* < 0.05) in the old group which were significantly increased up to 3.8-fold, 4.36-fold, 2.39-fold, 3.04-fold, and 3.27-fold, respectively. The expression of periostin (*P* < 0.05) in the old group was -12.7-fold compared with that in the young group, and the expression of kininogen 1 (*P* < 0.05) in the old group was 12.4-fold compared with that in the young group. Results of RT-PCR were consistent with the results of microarray data. The results are shown in Figure 7.

## 4. Discussion

Cellular senescence serves as an important disease-causing determinant [12, 17]. Senescence of NPCs with age is closely related to the change of IVD aging and degeneration [7, 10, 11]. In this study, we compared the biological functions and analysed the differentially expressed gene in young and old NPCs to uncover the potential therapeutic target during NPC senescence. This study has a significant value in clinic therapy of IVDD because the key regulated genes during the NPC senescence could be manipulated to reactivate the senescent NPCs. It would be unnecessary to isolate NPCs from the tissue by invasive operation, patients may just need to receive molecules to rejuvenate NPCs, and then, the self-repair procedure could be started.

To identify the surface phenotypes of isolated young and old NPCs, a variety of surface markers were adopted for detection according to previous studies [23–25]. CD24 is a glycosylphosphatidylinositol-anchored cell surface protein, which has been defined as a marker of healthy NPCs by Risbud et al. [23]. Tang et al. found a strong expression of CD24 in juvenile human NP tissue, and its expression would decline with age [26]. Although the species in this study was different with previous studies, we also found the expression of CD24 in old NPCs decreased significantly than that in young NPCs. This result indicated that CD24 may serve as a marker of NPC senescence. We further detected the expression of CD90, which is a cell-surface-anchored glycoprotein found in many kinds of stem/progenitor cells [27]. In the present study, CD90 was highly expressed in both young and old NPCs. However, Tang et al. found that CD90 is only expressed in AF cells and may serve as a non-NP marker in rats [14]. We considered that this may be due to the different isolation method used in their study. Previous study of Molinos et al. found that NPCs highly express CD29 and Brachyury with a low expression of CD34, CD45, and CD146 [24]. In our study, the expression of CD34 and CD45 was lower than 5% in both the young and old groups, which indicated no contamination of hematopoietic-lineage cells. Both young and old NPCs could highly express CD29 (>95%), and its expression discovered no difference in young and old NPCs, which was consistent with the previous study [24].

Then, we compared the proliferation and migration capabilities of young and old NPCs. An age-related decline in the growth kinetics was reported for NPCs before. Jeong et al. found that human NPCs from young patients have a higher proliferation ability and less SA-*β*-gal staining percentage than old ones [28]. In line with the above-mentioned research, our study also showed a diminished proliferation capacity of old NPCs. We further investigate the migration capability between young and old NPCs, and results showed a declined migration ability in old NPCs than young NPCs. Thus, based on the combined above results, we propose that the declined proliferous and migratory capabilities of NPCs are two key processes of IVD aging.

In the present study, we further revealed the DEGs between young and old NPCs. Among the downregulated genes, periostin was the most significantly altered gene. Previous studies have shown that periostin could regulate the proliferation and differentiation of many kinds of cells such as periodontal ligament mesenchymal stem cells [29, 30], skeletal stem cells [31], and adipose-derived stem cell [32, 33]. Furthermore, periostin could interact with structural collagens, thereby influencing the mechanical structure of the ECM in a local tissue [34]. Egbert et al. found that periostin contributes to proper collagen function and is downregulated during skin aging, indicating an important role of periostin in the regulation of collagen function [35]. Previous research found that periostin is also being expressed in the human and rat IVD and has relevance to the IVDD because it binds to several ECM components such as fibronectin, tenascin, and collagen V and is related to the expression of several inflammatory cytokines such as IL-4 and TNF-*α* [36]. However, Tsai et.al reported an increased expression of periostin in IVD cells during IVDD, which was contradicting with our results [37]. We consider that it is because their model represents rapid injury of IVD, thus stimulating periostin expression to regulate the structure of ECM. The reaction of cells to the change of environment might be declined with age, thus showing a downregulation of periostin of NPCs. Consistent with our hypothesis, Graja et.al reported that loss of periostin occurs in aging adipose tissue is closely associated with the age-related alterations of the adipose tissue extracellular matrix [32]. Moreover, Duchamp de Lageneste et.al discovered that the bone regenerative potential of skeletal stem cells in periosteum is determined by periostin [31]. Thus, methods that upregulate the expression of periostin might reverse the malfunction of NPCs during aging. Among the high expression genes, kininogen 1 was the highest one. Kininogen 1 has been detected in numerous pathophysiological conditions, such as arthritis [38] and inflammatory bowel disease [39]. Although there is no literature that reported the association of kininogen 1 with IVD aging, the relationship of kininogen 1 with aging in other tissues has been recognized [40, 41]. Besides, Dai et.al reported that cleaved kininogen could accelerate the onset of endothelial progenitor cell senescence by activating the ROS-p38 kinase-p16INK4a signaling cascade [42]. Therefore, kininogen 1 might have a similar effect on NPC senescence.

Functional annotation of upregulated genes was conducted by GO and KEGG pathway analysis. Interestingly, the top 5 enrichments of GO BP included response to lipopolysaccharide (GO BP:003249), negative regulation of cell proliferation (GO BP:0008285), and response to hypoxia (GO BP:0001666). Lots of literatures reported that lipopolysaccharide could induce inflammatory response of IVD; thus, the biological processes in response to lipopolysaccharide indicated an important role of inflammation in NPC senescence [43]. Besides, hypoxia and inflammation are two important characteristics of the environment of degenerated IVD [44]; thus, these results highlighted a vital role of a local environment in NPC senescence. Additionally, the most significant signaling pathways of upregulated genes included the TNF signaling pathway (KEGG:rno04668), PI3K-Akt signaling pathway (KEGG:rno04151), and pathways in cancer (KEGG:rno05200). The TNF signaling pathway has been widely recognized as a vital regulator of the inflammatory cascade during IVDD [45, 46]. Thus, our results showed that the biological function of old NPCs might been influenced by the harsh conditions existing in the microenvironment of IVD. Among the downregulated genes, GO functional enrichment of BP is mainly enriched at cell adhesion (GO BP:0007155), cell-matrix adhesion (GO BP:0007160), and endodermal cell differentiation (GO BP:0035987). Pathway enrichment analysis is mainly at ECM-receptor interaction (KEGG:rno04512) and focal adhesion (KEGG:rno04510). Interestingly, these results were consistent with our cellular experiment results which showed a declined migratory capability of old NPCs. Therefore, these results indicated a decreased migratory capability of old NPCs.

We further analysed the hub genes in the PPI network by 6 calculation methods. All derived 5 hub genes (Cxcl1, Egr1, Ptgs2, Fos, and Igf1) were upregulated in the old group. We further conducted real-time PCR to verify the results of microarray. Results showed a consistent expression trend with microarray. Cxcl1, Egr1, and Ptgs2 have been reported to be existing in IVD tissues and revealed a close relationship with the immune response and inflammation. [47–49] Fos is a well-studied oncogene. It was reported that Fos existed in the herniated disc tissue instead of the healthy disc [50]. Besides, a recent study had shown that Fos could regulate the transcription of Sox9 and that the cfos-Sox9 axis is critical for the role of cfos in the induction of chondroblastic Osteosarcoma [51]. Considering that SOX-9 is also an important marker of nucleus pulposus cells, the role of cfos in the regulation of NPCs during aging is needed to be deeply investigated. Igf1 is a growth factor known to activate matrix metabolism. Igf1 has also been found to have a close relationship with IVDD [52]. A previous study has described that the responsiveness of chondrocytes to IGF-I decreased with age [53]. Okuda et.al found that the increased expression of IGF-I binding proteins (IGFBPs) and downregulation of IGF-I receptor might be the two key mechanisms for the aging-related nonresponsiveness to IGF-1 [54]. Therefore, we hypothesized the upregulated expression of IGF-1 might be the compensation feedback in old NPCs. We also performed the GO and KEGG analysis of derived hub genes. Results showed that the TNF signaling pathways in response to lipopolysaccharide were enriched, which is consistent with the GO and KEGG results of upregulated genes.

The three most significant submodules of DEGs were extracted from the PPI network with MCODE scores of ≥4. After GO functional and KEGG pathway enrichment analyses of the DEGs in the highest scored modules, the genes in this module were mainly associated with the cellular response to organic substance (GO:0071310), skeletal muscle cell differentiation (GO:0035914), and inflammatory response (GO:0006954). The pathways were enriched in the TNF signaling pathway (rno04668), and PI3K-Akt signaling pathway (rno04151), which further illustrated the importance of these two pathways during NPC aging.

There were several limitations in this study. First, numerous RNA probes were detected in the microarray analysis and this limited the validation of the gene chip results. Therefore, we only interpreted the results based on previous studies and our interests. It was reported that NP consists of a mixture of small chondrocyte-like mesenchymal cells and larger notochordal-derived cells [26]. In mature NP tissues, notochordal cells gradually disappeared, replaced by smaller fibrochondrocyte-like cells [8]. However, the harvested cells in this study were mainly fibrochondrocyte-like cells in both the young and old groups, which was different with the cellular morphology of notochordal cells (characterized by lots of vacuoles). The culture method in this study was a monolayer with normoxic condition, which may be difficult to preserve the phenotype of notochordal cells due to the phenotype of notochordal cells that would quickly disappear after in vitro culture. Since the optimal culture condition for notochordal cell phenotype maintenance is still under investigation, our study may only represent a part of the mechanism of NPC senescence. Another limitation exists in the normoxic culture condition. Hypoxia is one of characteristics of IVD. Previous studies shown that the gene expression of NPCs may be altered due to the normoxic culture condition [55]. Thus, hypoxia and 3 dimensional cultures may be more suitable to maintain the phenotype of NPCs.

## 5. Conclusions

Taken together, we discovered that the old NPCs showed declined proliferation and migration abilities. Furthermore, we identified a series of molecular pathways that contribute to NPC aging and degeneration through microarray analysis. Further studies will be needed to elucidate downstream mechanisms of potential targets. An understanding of the mechanisms underlying these aging processes may lead to a novel breakthrough in the prevention and treatment of disc aging and aging-related degeneration.

## Figures and Tables

**Table 1:** Primers used for real-time PCR.

Gene name	Forward sequence 5′-3′	Reverse sequence 5′-3′
Fos	CAAACCGACCTACTGTCCC	ACCAACAACCTTGTCGTCATAT
Egr1	GCAACACTTTGTGGCCTGAA	GAGTTGGGACTGGTAGGTGT
Cxcl1	AAATGGTGAAGGTCGGTGTGAAC	CAACAATCTCCACTTTGCCACTG
Igf1	GCACTCTGCTTGCTCACCTTTA	TCCGAATGCTGGAGCCATA
Ptgs2	TTCGGGAGCACAACAGAGTG	TGAAGTGGTAACCGCTCAGG
Kininogen 1	ATGGTCCCGACTGTGAAATGCCAAG	CTATCTCTGGGTAGTCTGCTTTACAC
Periostin	ACAAGCCAACAAAAGGGTTCA	ACGGCCTTCTCTTGATCGC
GAPDH	GGCATCCTGGGCTACACT	CCACCACCCTGTTGCTGT

**Table 2:** The top 10 upregulated genes.

Gene names	Fold change	*P* value
Kininogen 1	25.06	2.00117*E*-05
Lipocalin 2	15.25	4.43512*E*-05
EGF-containing fibulin-like extracellular matrix protein 1	12.85	0.000583062
Collagen 14a1	12.81	3.332*E*-05
Phospholipase A2	10.35	7.85477*E*-05
Interferon-induced transmembrane protein 1	8.89	0.000557976
LOC100125362	8.48	8.31466*E*-06
Carboxypeptidase X	7.57	1.60859*E*-05
Complement component 6	7.48	5.87991*E*-05
Inhibitor of DNA binding 1	7.48	0.000073019

Filter criteria for significant difference gene: ∣fold change∣ > 1.5, *P* value < 0.05.

**Table 3:** The top 10 downregulated genes.

Gene names	Fold change	*P* value
Periostin	-24.05	1.60411*E*-06
Neuronal regeneration-related protein	-8.77	5.64411*E*-06
Dermatopontin	-6.57	8.44669*E*-05
Integrin alpha 4	-6.44	0.000120932
Syntaxin-binding protein 5-like	-6.12	5.20189*E*-07
Fibulin 2	-6.11	1.64021*E*-05
Dihydropyrimidinase-like 3	-6.09	0.000253521
Calponin 1	-6.03	0.000115957
Sphingosine-1-phosphate receptor 3	-5.72	9.20555*E*-05
Gliomedin	-5.30	0.000144277

Filter criteria for significant difference gene: ∣fold change∣ > 1.5, *P* value < 0.05.

**Table 4:** List of top 5 terms of upregulated genes enriched by GO and KEGG analysis.

Terms	Count	-log10 (*P* value)
KEGG:rno04668:TNFsignaling pathway	15	4.863619311
KEGG:rno04151:PI3K-Aktsignaling pathway	27	4.249211392
KEGG:rno05200:Pathways in cancer	29	3.912805981
KEGG:rno04630:Jak-STAT signaling pathway	14	3.272521081
KEGG:rno04512:ECM-receptor interaction	10	2.566175661
GO BP:0032496~response to lipopolysaccharide	29	7.485884419
GO BP:0014070~response to organic cyclic compound	28	7.172760304
GO BP:0008285~negative regulation of cell proliferation	33	6.863888026
GO BP:0001666~response to hypoxia	27	6.668325051
GO BP:0050680~negative regulation of epithelial cell proliferation	13	5.878814283
GO MF:0004866~endopeptidase inhibitor activity	9	5.741812795
GO MF:0001077~transcriptional activator activity, RNA polymerase II core promoter proximal region sequence-specific binding	24	5.695518301
GO MF:0005509~calcium ion binding	39	3.672674033
GO MF:0008134~transcription factor binding	23	3.591418924
GO MF:0043565~sequence-specific DNA binding	33	3.418128712
GO CC:0005615~extracellular space	99	16.94638876
GO CC:0070062~extracellular exosome	135	9.756235253
GO CC:0009986~cell surface	48	8.51199254
GO CC:0005578~proteinaceous extracellular matrix	25	6.200499673
GO CC:0031012~extracellular matrix	25	6.169491531

**Table 5:** List of top 5 terms of down regulated genes enriched by GO and KEGG analysis.

Terms	Count	-log10 (*P* value)
KEGG:rno04512:ECM-receptor interaction	12	6.305467082
KEGG:rno04510:Focal adhesion	16	5.283730936
KEGG:rno04151:PI3K-Akt signaling pathway	18	3.918980827
KEGG:rno04974:Protein digestion and absorption	8	2.964674869
KEGG:rno04024:cAMP signaling pathway	11	2.565239027
GO BP:0007155~cell adhesion	19	5.677119186
GO BP:0035987~endodermal cell differentiation	6	3.820807813
GO BP:0001503~ossification	9	3.307311242
GO BP:0007160~cell-matrix adhesion	8	3.272206365
GO BP:0010628~positive regulation of gene expression	17	2.767181699
GO MF:0005201~extracellular matrix structural constituent	7	3.709602001
GO MF:0005509~calcium ion binding	28	3.596212048
GO MF:0003735~structural constituent of ribosome	18	2.633105261
GO MF:0044325~ion channel binding	9	2.592276912
GO MF:0005178~integrin binding	8	2.584636716
GO CC:0031012~extracellular matrix	28	12.71649934
GO CC:0005604~basement membrane	16	9.863156842
GO CC:0005615~extracellular space	53	6.939058168
GO CC:0005578~proteinaceous extracellular matrix	20	6.645158563
GO CC:0005925~focal adhesion	25	6.484468766

**Table 6:** KEGG and GO analysis of the highest scored module genes.

Terms	-log10 (*P* value)	Count
GO BP:0071310~cellular response to organic substance	4.428041285	4
GO BP:0035914~skeletal muscle cell differentiation	4.06731104	4
GO BP:0006954~inflammatory response	4.016795968	6
GO BP:0009611~response to wounding	3.559234434	4
GO BP:0032496~response to lipopolysaccharide	3.016316347	5
KEGG:rno04668:TNF signaling pathway	3.913584453	5
KEGG:rno04151:PI3K-Akt signaling pathway	1.320427527	4
KEGG: rno04060:Cytokine-cytokine receptor interaction	1.074017917	3

**Table 7:** The hub genes were analysed by different topological algorithms in the protein-protein interaction network.

Topological algorithm	Top 20 genes were ranked by score
Maximal Clique Centrality (MCC)	Btg2, Clu, Csf1, Cx3cl1, Cxcl1, Cxcl5, Dusp1, Egr1, Egr2, Fos, Ier2, Igf1, Junb, Kng1, Kng2, Nfkbiz, Penk, Ptgs2, Serping1, Zfp36
Degree	A2m, Acta2, Clu, Col3a1, Csf1, Cxcl1, Cxcl5, Dusp1, Egr1, Fos, Igf1, Il7, Kitl, Kng1, Kng2, Postn, Ptgs2, Socs3, Vcam1, Vcan
Edge Percolated Component	Cd68, Clu, Cp, Csf1, Cx3cl1, Cxcl1, Cxcl5, Egr1, Fos, Igf1, Il7, Kitl, Kng1, Kng2, Nfkbiz, Penk, Ptgs2, Serpina3n, Socs3, Vcam1
Maximum Neighborhood Component	Acta2, Clu, Col3a1, Csf1, Cx3cl1, Cxcl1, Cxcl5, Dusp1, Egr1, Fos, Igf1, Il7, Kitl, Kng1, Kng2, Nfkbiz, Postn, Ptgs2, Serpina3n, Vcam1
BottleNeck	A2m, Acta1, Cd68, Cfh, Cxcl1, Dusp1, Egr1, Eng, Folr2, Fos, Igf1, Il7, Jag1, Kitl, Mcam, Negr1, Postn, Ptgs2, Timp3, Vcam1
EcCentricity	Acta2, Cd68, Csf1, Cx3cl1, Cxcl1, Egr1, Eng, Fgf10, Fgf9, Fgfr2, Fos, Igf1, Junb, Lyz2, Mmp12, Myh11, Ptgs2, Serping1, Smad9, Vcam1
Common genes of 6 topological algorithms	Cxcl1, Egr1, Fos, Igf1, Ptgs2

**Table 8:** KEGG and GO analysis of hub gene.

Terms	-log10 (*P* value)	Count
KEGG:rno04668:TNF signaling pathway	2.937464369	3
KEGG:rno05200:Pathways in cancer	1.8358872	3
KEGG:rno04913:Ovarian steroidogenesis	1.543625084	2
KEGG:rno05140:Leishmaniasis	1.435827789	2
KEGG:rno05132:Salmonella infection	1.375008637	2
GO BP:0032496~response to lipopolysaccharide	4.797953003	4
GO BP:0051384~response to glucocorticoid	3.469546007	3
GO BP:0042127~regulation of cell proliferation	2.980755828	3
GO BP:0014070~response to organic cyclic compound	2.85105501	3
GO BP:0042493~response to drug	2.282716935	3

## Data Availability

The data used to support the findings of this study are included within the article and the supplementary materials. The gene microarray datasets had been uploaded to the NCBI Gene Expression Omnibus (GEO) and can be accessed via GEO Series accession [GEO:GSE126883] (https://www.ncbi.nlm.nih.gov/geo/query/acc.cgi?acc=GSE126883). Other related data generated and/or analysed during the current study are available from the corresponding authors upon reasonable request.

## Abbreviations

NP: Nucleus pulposus
IVD: Intervertebral disc
RT-PCR: Real-time polymerase chain reaction
SA-*β*-gal: Senescence-associated *β*-galactosidase
FITC: Fluorescein isothiocyanate
PE: Phycoerythrin
EPC: Edge Percolated Component
MCC: Maximal Clique Centrality
MNC: Maximum Neighborhood Component.

## Supplementary Materials

Supplementary Materials Heat map and hierarchical clustering of DEG profile comparison between the young and old NPCs. Red colour indicates high expression, and green colour indicates low expression. Every column represents a tissue sample, and every row represents an mRNA probe. Z641, Z643, and Z642 were samples in the old group; Z644, Z645, and Z646 were samples in the young group.
