# Supplementary material for: Identification of Aberrantly Expressed Genes during Aging in Rat Nucleus Pulposus Cells
Source: Stem Cells Int. 2019 Jul 10;2019:2785207. doi: 10.1155/2019/2785207 (PMC6652086; doi:10.1155/2019/2785207)
Supplement: Supplementary Materials — Heat map and hierarchical clustering of DEG profile comparison between the young and old NPCs. Red colour indicates high expression, and green colour indicates low expression. Every column represents a tissue sample, and every row represents an mRNA probe. Z641, Z643, and Z642 were samples in the old group; Z644, Z645, and Z646 were samples in the young group. [file 2785207.f1.zip › supplementary material-RNA intergrate_SCI_2782874.docx]

**Supplementary materials-RNA Integrity**

The identification results of the purity and integrity of extracted RNA. Results of Thermo NanoDrop 2000 represented the purity of RNA, while the results of RNA Integrity Number (RIN) indicating the acquired RNA were integrated. These two results indicated acquired RNA meet the requirements for the sequencing experiments. The two pictures were the sample electropherograms analyzed on the Agilent 2100 bioanalyzer.

| number | Sample label | Sample name | Thermo NanoDrop 2000 | | 2100 Result | | result |
| --- | --- | --- | --- | --- | --- | --- | --- |
|  |  |  | density(ng/μL) | A260/A280 | RIN | 28S/18S |  |
| 1 | Z641 | Young 1 | 407.1 | 1.91 | 10 | 2.1 | qualified |
| 2 | Z642 | Young 2 | 422 | 1.89 | 9.9 | 2.1 | qualified |
| 3 | Z643 | Young 3 | 472 | 1.91 | 10 | 2.1 | qualified |
| 4 | Z644 | Old 1 | 458.4 | 1.96 | 10 | 2.1 | qualified |
| 5 | Z645 | Old 2 | 380 | 1.92 | 9.9 | 2.1 | qualified |
| 6 | Z646 | Old 3 | 344.9 | 1.93 | 9.8 | 2.1 | qualified |

**
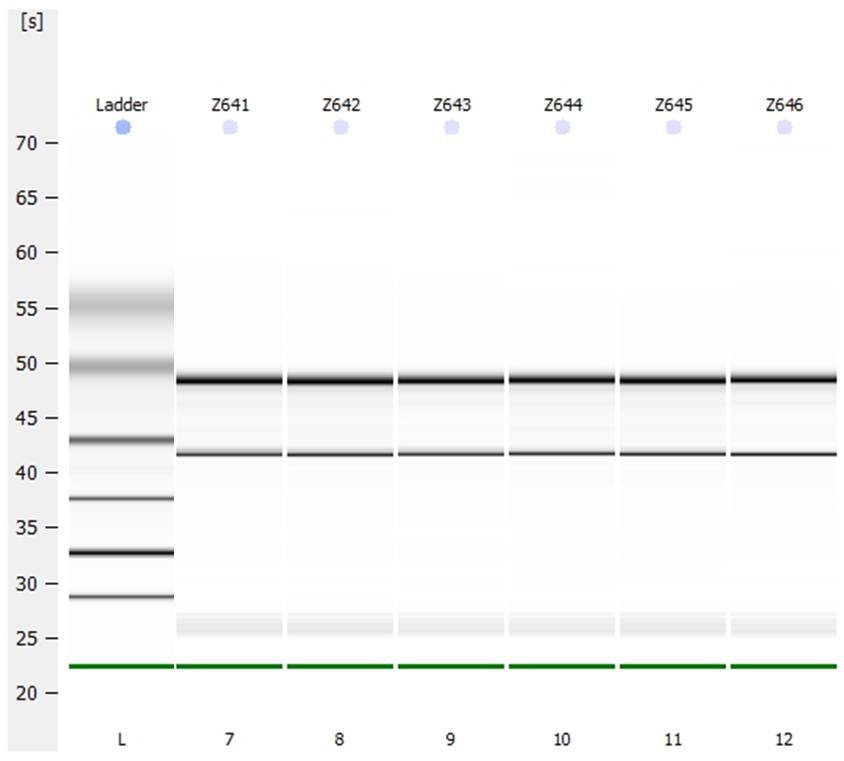
**

**
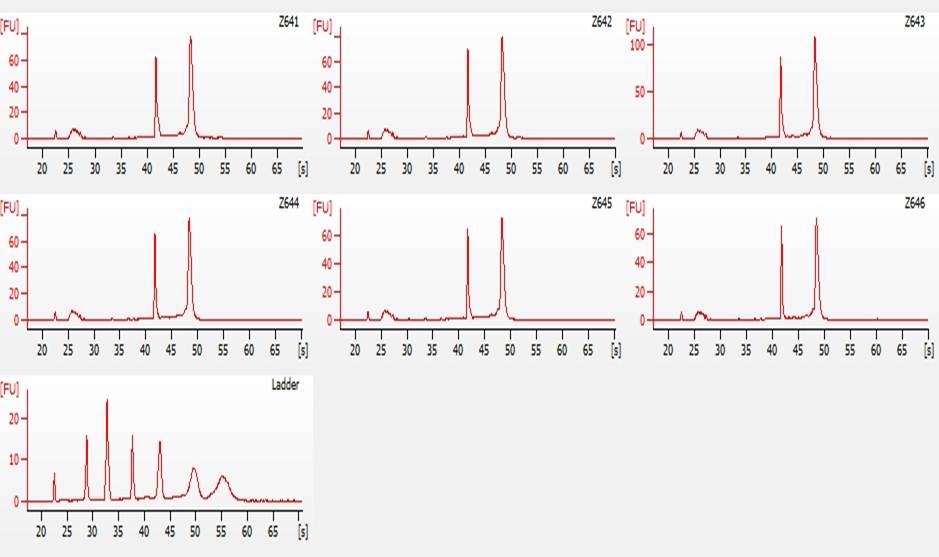
**
