# Supplementary figures and images for: Identification of Aberrantly Expressed Genes during Aging in Rat Nucleus Pulposus Cells
Source: Stem Cells Int. 2019 Jul 10;2019:2785207. doi: 10.1155/2019/2785207 (PMC6652086; doi:10.1155/2019/2785207)

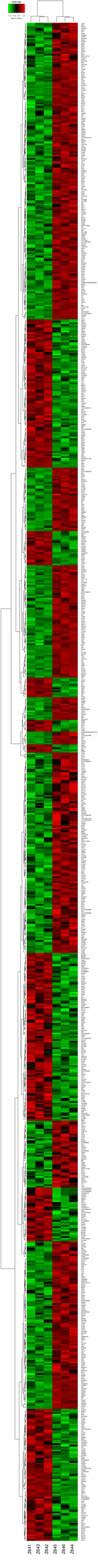

Supplement: Supplementary Materials — Heat map and hierarchical clustering of DEG profile comparison between the young and old NPCs. Red colour indicates high expression, and green colour indicates low expression. Every column represents a tissue sample, and every row represents an mRNA probe. Z641, Z643, and Z642 were samples in the old group; Z644, Z645, and Z646 were samples in the young group. [file 2785207.f1.zip › Supplementary material-Heatmap_SCI_2782872.pdf]
